# Effects of Physical Activity Program on Body Composition, Physical Performance, and Neuromuscular Strategies during Walking in Older Adults with Sarcopenic Obesity: Randomized Controlled Trial

**DOI:** 10.3390/healthcare11162294

**Published:** 2023-08-14

**Authors:** Hamza Ferhi, Sabri Gaied Chortane, Sylvain Durand, Bruno Beaune, Sébastien Boyas, Wael Maktouf

**Affiliations:** 1 Research Laboratory (LR23JS01) « Sport Performance, Health & Society », Higher Institute of Sport and Physical Education of Ksar Saîd, University of “La Manouba”, Tunis 2010, Tunisia; sabrigaied1@gmail.com; 2 Laboratory “Movement, Interactions, Performance” (UR 4334), Department of Sport Sciences, Faculty of Sciences and Technologies, Le Mans University, 72000 Le Mans, France; sylvain.durand@univ-lemans.fr (S.D.); bruno.beaune@univ-lemans.fr (B.B.); sebastien.boyas@univ-lemans.fr (S.B.); 3 Bioengineering, Tissues and Neuroplasticity, UR 7377, Faculty of Health, University of Paris-Est Créteil, 8 rue du Général Sarrail, 94010 Créteil, France; wael.maktouf@u-pec.fr

**Keywords:** age, obesity, gait, physical activity, neuromuscular system, sarcopenia

## Abstract

The potential impact of a specific physical activity program on biomechanical gait parameters and neuromuscular strategies around the ankle joint in older adults with sarcopenic obesity (SO) remains largely unexplored. The objective of this study was to investigate the effectiveness of a 24-week posture, strengthening, and motricity (PSM) program on improving neuromuscular strategies and biomechanical gait parameters in older adults with SO. 40 participants were randomly assigned to either the trained group (TG) and the control group (CG). Only the TG received the PSM program. Standardized evaluations were performed before and after the intervention, including walking tests on an instrumented gait analysis treadmill to evaluate biomechanical gait parameters and EMG activity of ankle muscles. After the PSM program, TG exhibited an increase in comfortable walking speed (+80%, *p* < 0.001) and step length (+38%, *p* < 0.05). Moreover, TG demonstrated a reduction in CoP velocity (−26%, *p* < 0.01). These gait modifications were associated with decreased muscle activity during the different gait phases (*p* < 0.05). The PSM program effectively improved gait and neuromuscular capacities in older adults with SO. Notably, these results shed light on the remarkable trainability of neuromuscular capacities in older adults with SO, despite the adverse effects of aging and obesity.

## 1. Introduction

The rising prevalence of obesity in older adults is a global concern, as it is accompanied by a natural increase in fat mass and decrease in muscle mass and strength, resulting in sarcopenic obesity (SO) [1,2,3]. The synergistic impact of obesity and muscle impairment heightens the risk of developing multiple health outcomes, including functional limitations and injury risk [4,5]. The associated muscle impairment may cause functional limitations in activities of daily living, particularly walking [6,7]. Furthermore, individuals with large body sizes but disproportionately low muscle strength are at a higher risk of both disability and future disability development [3,8]. Therefore, implementing tailored physical activity programs that suit older adults with SO is critical in preventing falls [9], enhancing mobility, and promoting healthy aging among obese older adults [10].

Aging is associated with a decline in a variety of neural, hormonal, and environmental trophic signals to muscle [11,12,13,14]. The loss of muscle mass and mass-specific strength is accelerated by a variety of factors, including physical inactivity [15], hormonal changes [16], pro-inflammatory state [17], malnutrition [18], and loss of alpha-motor units in the central nervous system [19,20]. These factors negatively alter functional capacities, postural control, and walking [21,22], limit autonomy, and increase the risk of falls [23,24].

Research has demonstrated that obese individuals may experience difficulties in performing simple motor tasks with sufficient velocity, and they often exhibit greater instability during walking [25,26]. In addition, spatiotemporal gait parameters, such as speed, cadence, and stride, are significantly lower in obese individuals [27,28,29]. These alterations are associated with multiple adverse consequences for skeletal muscle, including inflammation [30], oxidative stress [31], and insulin resistance [32]. Fatty infiltration of skeletal muscle is also associated with reduced strength, functional status, and muscle dysfunction, decreased contractility and motor unit recruitment, and interference in normal cellular signaling [33].

In addition to the above studies, there is growing evidence for the synergistic impact of sarcopenia and obesity on gait and mobility in older adults [6,34,35,36]. In fact, obesity presents additional constraints to age-related postural control deteriorations [37,38,39], and increases risk of falls [40]. Recently, Maktouf et al. [34] showed that obese older adults had lower vertical ground reaction force (GRFv), higher center of pressure (CoP) velocity, shorter and wider stride, and spent more time in support phase. These gait alterations were associated with higher ankle muscle activity. These findings underscore the significance of addressing sarcopenia and obesity as interdependent yet distinct factors while devising interventions to enhance gait and mobility in older adults. Moreover, the impact of obesity on gait parameters may differ between adults and older individuals, emphasizing the need to differentiate between these age groups when designing interventions to enhance functional capacities.

Many studies have indicated that physical activity is one of the most effective non-pharmacological interventions for the management of older adults with SO [41,42,43]. Various studies have shown that physical activity can lead to improvements in body composition [41,42], muscle mass, strength [41,42,43,44], and physical performance [45,46,47,48]. However, no prior investigations have explored the effects of a physical activity regimen emphasizing posture, strengthening, and motricity exercises (PSM) on neuromuscular strategies, CoP displacements, and kinematic and kinetic gait parameters in older adults with SO. Furthermore, it is important to address some limitations observed in previous studies. These include the lack of information on the methodology for setting up the physical activity protocol, the program’s progressiveness, the quality and intensity of exercises to be performed, and the quantification of each session based on individual feedback. These limitations underscore the need for more comprehensive studies that develop effective physical activity interventions tailored to the specific needs and capacities of older adults with SO.

The main objectives of this study were twofold. Firstly, we aimed to assess the effectiveness of the PSM program on improving body composition and physical performance among older adults with SO. Secondly, we sought to investigate the effects of the PSM program on neuromuscular strategies during walking, ankle muscle strength, and gait parameters. We hypothesize that the PSM program changes body composition and improves physical performance in older adults with SO. Furthermore, we anticipate that the PSM program bolsters ankle muscle strength, modifies neuromuscular strategies during walking, and optimizes both kinematic and kinetic gait parameters in older adults with SO.

## 2. Materials and Methods

### 2.1. Study Design

**Figure 1:**
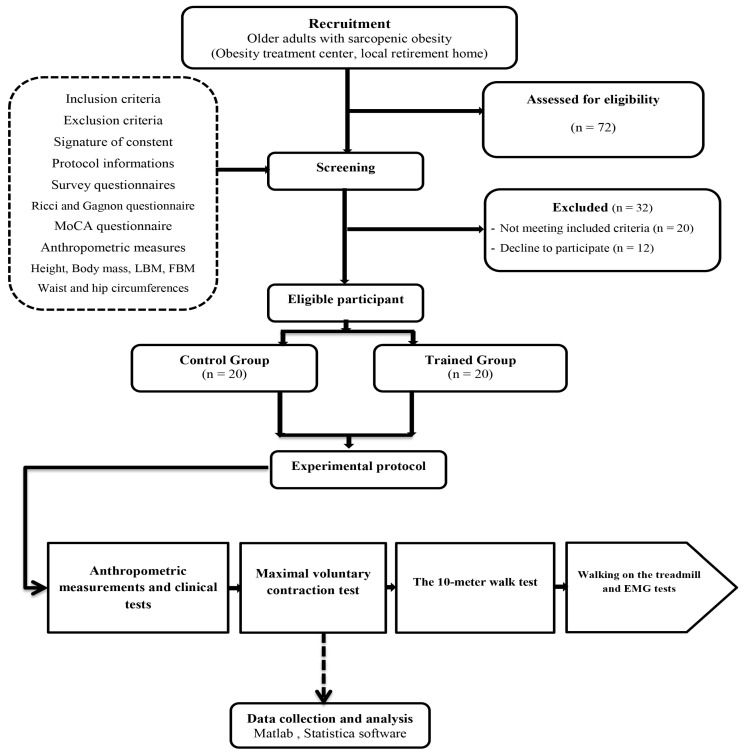
Flow diagram illustrating the experimental procedure design.

Our study, a single-blinded multicenter randomized controlled trial, strategically placed participants into either a trained group (TG) or a control group (CG), as shown in Figure 1. The CG continued their regular daily activities and underwent evaluations pre- and post-study. Meanwhile, the TG participated in a structured 24-week PSM program, including two weekly sessions. The recruitment process for this study followed a systematic approach that involved a three-week recruitment period, a one-week screening phase, and three weeks of experimental testing before and after the intervention. Comprehensive assessments were conducted both before and after the intervention, utilizing a standardized protocol that included five specific assessments. These assessments comprised anthropometric measurements, walking and treadmill tests, maximal voluntary contraction tests, and thorough clinical evaluations.

Our study was carried out in compliance with the Helsinki Declaration. A local ethics committee approved the study protocol, patient information letter, and informed consent form. Our research was registered in the Pan African Clinical Trials Registry under the registration number PACTR202306912191110. This study and intervention are reported using the Consolidated Standards of Reporting Trials (CONSORT) and Template for Intervention Description and Replication guidelines (TIDieR) [49,50].

### 2.2. Participants 

#### 2.2.1. Eligibility Criteria

Participants were recruited from various obesity care centers located across the Tunis region, employing a recruitment strategy that combined direct clinic recruitment and leaflet distribution from 1st February to 31st October 2022. Participants had to meet specific criteria: having a BMI > 30 kg/m^2^, a Handgrip force (HF) < 17 N, gait speed < 1.0 m/s, being over 65 years old, having the ability to verbally communicate with the experimenters, and being physically independent. Exclusion criteria included the presence of neurological or cognitive impairments, severe cardiovascular problems, severe musculoskeletal deformities or injuries of the lower limb, co-morbidities, or chronic diseases, medication use that could interfere with testing, and a Montreal Cognitive Assessment (MoCA) test score of <26. These criteria were verified through a comprehensive survey questionnaire and reviewed by the medical staff at the respective facilities. Participants’ physical activity levels and cognitive scores were assessed using the Ricci and Gagnon [51] and the MoCA tests [52]. Written consent to participate in the protocol was also signed directly by patients.

#### 2.2.2. Sample Size

The sample size was calculated using the freeware G*Power (version 3.1.9.4) [53]. The ANOVA test was predefined for power analysis. The estimation was based on predefined control of type I error (alpha = 0.05) and Type II error (beta = 0.60), with a moderate level of estimated effect size (r = 0.35). Under these settings, 40 participants were required as the minimum sample size.

#### 2.2.3. Randomization Procedure

The randomization list was generated by a computer algorithm by an independent statistician from our partnering institution, ensuring that the study team remained impartial. The unique randomization number for each participant was obtained by the evaluating investigator (who was blinded to the treatment assignment) at the end of the screening process. This number was then electronically sent to the coordinating investigator at each center, who was unblinded to the assignments. Participants were randomized into one of two groups: the CG who continued with their usual activities and the TG who undertook the 24-week PSM program. To account for possible center effects, randomization was stratified by center, ensuring a balanced representation of participants from each center in both the CG and TG.

### 2.3. Intervention: PSM Program

**Figure 2:**
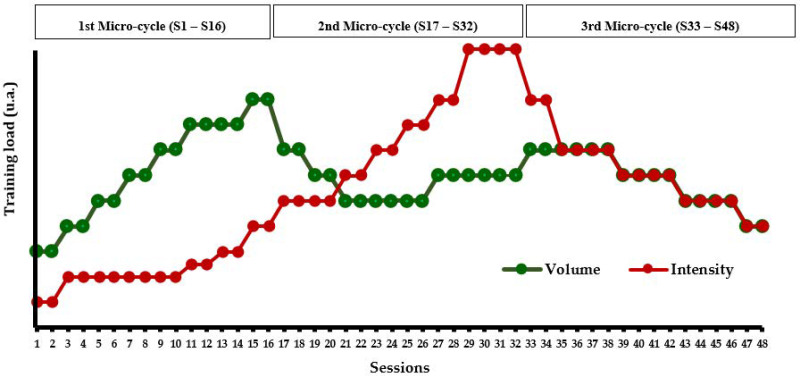
Fluctuations in volume and intensity throughout the 48-session course of the PSM program.

**Figure 3:**
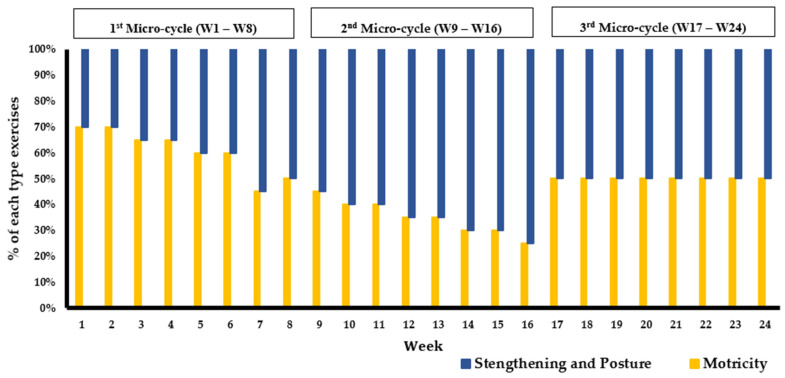
Weekly exercise quantity (duration) over 24 weeks of the PSM program. Note: the PSM program was divided into 3 micro-cycles based on the type of exercises. The first cycle mainly focused on motor skills exercises (50% to 70% of the session), the second on balance and muscle strengthening exercises, and the third cycle offered all types of exercises in a balanced way. The quantity of the different types of exercise was regulated every week.

The PSM program is described based on a study conducted by Maktouf et al. [44] and adheres to guidelines provided by the TiDieR, which encompass the following 12 items:

1. ***Name*:** The PSM program
2. ***Why*:** The PSM program aimed to enhance physical performance, walking capacity in older adults with SO.
3. ***What (Materials)*:** The PSM program employed various physical materials to aid in the performance of the exercises; chairs for exercises such as sit-to-stands, balls for coordination and balance training, a stopwatch for timing exercises, markers, slats, and cups for creating obstacles or guiding movement paths, hoops, elastic bands, and weighted bags to increase resistance and challenge in the exercises.
4. ***What (Procedures)*:** The PSM program spanned 24 weeks, including two 60-min sessions per week, for a total of 48 sessions. Each session includes a warm-up (10 min), motor skill exercises (duration based on the pre-set training volume), strengthening/posture exercises (duration based on the pre-set training volume), and a cool-down phase (5 min). Motor Skill exercises form an interactive segment involving dynamic movements such as interactive walking, where participants maintain eye contact as they walk towards each other or retreat to their original positions. These exercises also include various obstacle courses that require participants to navigate through specified areas, stepping over objects like cups or hoops without making contact. Additionally, participants engage in weight-fetching drills, which require rising from a seated position, navigating obstacles, retrieving weighted bags, and returning to the start point. To add a layer of complexity and competitive spirit, these exercises are varied by increasing the number of objects, timing tasks, or introducing competitive elements among groups. Simultaneously, strength training exercises focus on enhancing various muscle groups. Participants execute a wide array of movements, including ankle dorsiflexion, plantar flexion, and exercises that emphasize upper body and postural muscles such as arm flexion/extension and lumbar muscle strengthening. Resistance training is also integrated using weights and balls, adding an additional challenge to the exercises. Progressive challenges are introduced by adjusting the speed of execution, altering arm positions, and incrementally adding weights to amplify the difficulty level. The program also incorporates posture exercises designed to enhance the proprioceptive system and improve balance. Participants engage in exercises such as balancing on unstable surfaces and resisting mild pressure exerted on various body parts by a partner. They also perform exercises like traversing obstacles with added weights, stimulating their balance and postural control. Multi-directional pressure resistance exercises further enhance balance, sometimes incorporating unexpected pushes to present an added layer of challenge.
5. ***Who*:** By a kinesiologist specialized in adapted physical activity.
6. ***How*:** In collective, face-to-face sessions.
7. ***Where*:** In the fitness hall of each respective structure.
8. ***When and how much*:** The PSM program begins on the 21st of March and concludes on the 21st of September 2022, spanning a total of 24 weeks. Each week includes two sessions, making a cumulative total of 48 sessions. Each session is 60 min long. The design of all exercise types within the program was based on each session’s specific training load (see item 9) and on pre-set training intensity and volume for each session (see item 11). The regimen for each exercise type involved anywhere between 1 to 5 sets, with the repetitions for each set ranging from a minimum of 3 to a maximum of 15.
9. ***Tailoring*:** The variation in training load was determined after each session, based on the rating of perceived exertion scale (RPE) [54]. The RPE scale ranges from 0 (no difficulty) to 10 (extremely difficult), and the training load of the group was calculated by multiplying the session’s RPE score by its duration (e.g., for a group with an average RPE score of 6 in a 60-min session, the training load would be equal to 360 arbitrary units (a.u.). This method ensures the training’s efficiency, especially regarding the progression of solicitation, and allows for the monitoring of a possible overtraining syndrome. When the training load exceeded 300 a.u. (equivalent to 5 × 60 min), it was maintained for the next session. When the training load was below 300, the number of series and repetitions was increased by 25% in the next session.
10. ***Modifications*:** Adjustments were made to the exercises in each session, with an increase in the number of series or repetitions based on the training load. The difficulty was also progressively enhanced by adding obstacles, setting a time limit for task completion, and other means to ensure gradual progression and increasing solicitation.
11. ***How well (planned)*:** The program’s progressiveness was based on two aspects: a quantitative aspect related to the training load (volume/intensity) and a qualitative aspect related to the type of exercises (muscle strengthening, balance, and motor skills exercises). According to the quantitative aspect, the PSM program was structured into 3 micro-cycles of 16 sessions each: the first micro-cycle focused on volume, the second on intensity, and the third micro-cycle aimed to balance both volume and intensity (Figure 2). According to the qualitative aspect, the PSM program was divided into 3 micro-cycles based on the type of exercises. The first cycle mainly focused on motor skills exercises (i.e., higher duration), the second on balance and muscle strengthening exercises, and the third cycle offered all types of exercises in a balanced way. The quantity of the different types of exercise was regulated every week (Figure 3).
12. ***How well (actual)*:** the TG attended all sessions.

### 2.4. Evaluation Protocol

The evaluations described below were conducted in an identical manner and at the same time of day by the same evaluating investigator before and after the PSM program.

#### 2.4.1. Anthropometric Measurements and Clinical Tests

Anthropometric measurements were taken, including height, waist circumference (measured at the midpoint between the 12th rib and the iliac crest), and hip circumference (measured at the widest part of the hip) using a tape measure. Body weight (BW) and fat body mass (FBM, %) were measured using an impedance-meter (Tanita; SC 240-Class III; Tanita Europe B.V., Amsterdam, The Netherlands). FBM and lean body mass (LBM) were calculated using the equations from [55]:

FBM = body fat (%) × body mass; and LBM = body mass − FBM

Then, participants were instructed to complete two walking trials in a 20-m corridor to determine their preferred and maximal gait speed (m/s). To eliminate the acceleration and deceleration phases from the analysis, only the speed between the 5th and 15th meters was measured. Finally, the senior fitness test was performed as assessment tools to measure the physical performance of participants and detect any functional limitations [56].

#### 2.4.2. Maximal Voluntary Contraction Test

Isometric contractions of the ankle plantar flexors (PFs) and dorsal flexors (DFs) muscles of the dominant leg, maximal voluntary contractions (MVC) were recorded using a dynamometer (Sauter FL1K; Type: Force Gauge; Sauter GmbH, Balingen, Germany). Participants were instructed to keep their back, buttock, and thigh in contact with the chair and their leg stretched horizontally while pushing with the tips of the foot on the dynamometer for PFs. For DFs, participants were asked to stand up, keep their ankle at 90° and push with the foot on the dynamometer [57]. To ensure stability, a strap was placed around the dominant leg and firmly secured to the foot plane during PF contractions. During each contraction, participants were strongly encouraged to provide maximal effort. Two trials were performed with a 1-min rest in-between, and the mean of the single maximal value of the two trials was taken for both MVC of PFs and DFs. Relative force (MVC/Body weight, N/kg) was calculated.

#### 2.4.3. Walking Test

Participants were familiarized with walking on an instrumented gait analysis treadmill (Zebris; FDM-T) for 5 min at their pre-calculated preferred walking speed previously measured, followed by a 2-min seated rest period. If participants experienced any difficulty in walking on the treadmill, they were given an additional 2 min of training. Subsequently, participants performed three 1-min trials, with each trial separated by a 5-min seated rest period. Data were collected for a period of 10 s, starting at the 10th second of each trial. The treadmill software 1.12 (Zebris, FDM-T) sampled the gait parameters at 100 Hz from the treadmill force plates. These parameters included spatiotemporal parameters, walking cycle phases, GRFv, CoP displacements. Spatiotemporal parameters such as step length, step width, and stride length were recorded. The software divided the walking cycle into support phase (SU) and swing phase (SW), consisting of the first double support (1st DS), a single support (SS), and the second double support (2nd DS). The absolute GRFv peaks were used to calculate the relative GRFv peaks (P1, P2, N/kg, respectively) by dividing the absolute value of the GRFv peak by the body mass. The CoP data were used to extract the CoP length during the SU and SS, the CoP anteroposterior position, and the CoP velocity during walking (cm/s).

#### 2.4.4. Electromyography Test

Electromyographic (EMG) data from ankle joint muscles were collected during MVC of PFs and DFs, and during a treadmill walking test using Trigno^®^ Wireless Biofeedback System (Delsys Inc., Natick, MA, USA). The EMG recording was synchronized with the treadmill data using a control device operating on the principle of a synchronization switch system (ON/OFF). The sensors composed of two pairs of silver bar contacts with 10 mm interelectrode spacing electrodes were placed on the gastrocnemius medialis (GM), soleus (SOL), and tibialis anterior (TA) of the dominant leg, conforming to the recommendations of SENIAM. The raw EMG signals were post-processed using Matlab software (Matlab R2013a, MathWorks, Natick, MA, USA). The data from 10 strides were collected for a period of 10 s, in the 10th second of each trial (i.e., the same period of the treadmill recording), and were band-pass filtered at 15–500 Hz through a second-order Butterworth digital filter to remove noise or movement interference [58]. The data were rectified and smoothed using root mean square analysis (RMS) with a 20-ms window [34] and calculated using the following equation [59]:

(1) $$\mathrm{RMS}\;(t_{0})=\sqrt{\frac{1}{T}\int_{t0-T/2}^{t0+T/2}{\left(\mathrm{EMG}\right)}^{2}dt,}\;\mathrm{where}\;T\;\mathrm{is}\;\mathrm{the}\;\mathrm{time}\;\mathrm{of}\;\mathrm{integration}$$

For the MVC tests, a moving window with a width of 20 ms was used to find the maximum RMS EMG activity resulting from the three efforts of MVC for each kind of contraction. Then, all RMS EMG data of walking on the treadmill test were normalized using the following equation for each muscle:

EMG RMS% = [(RMS EMG assessment/RMS EMG MVC) × 100%](2)

The normalized RMS of the GM (RMS GM), SOL (RMS SOL), and TA (EMG TA) of each walking cycle’s phase were used in the study.

### 2.5. Statistical Analysis

The statistical analyses were conducted using Statistica Software 13.0 (Software, Inc., Tulsa, OK, USA). The normality of the data distribution was checked using Kolmogorov–Smirnov tests. When the data distribution followed a normal distribution, paired t-tests were applied to compare the results of the same group before and after the PSM program. The independent samples t-test was also used to compare between the TG and CG before and after the PSM program. The relationships between the changes in gait parameters and the relative maximal force of the DFs and PFs, anthropometric parameters, and muscle activity of GM, SOL, and TA were evaluated using the Pearson’s correlation analysis. The data were expressed as means and standard deviations. The significance threshold was set at *p* < 0.05 for all results.

## 3. Results

### 3.1. Recruitment

In our study, we initially enlisted the participation of 72 volunteers through a recruitment strategy involving direct clinic recruitment and leaflet distribution in related centers. However, only 52 participants met our established eligibility criteria. Despite their initial inclusion, 12 individuals did not fully complete the study due to non-adherence to the protocol—7 from the CG and 5 from the TG. Several varied factors contributed to participant dropout, including the unfortunate passing of 2 participants, relocation to other centers for 3 individuals, health-related hospitalizations such as stroke, hip fracture, and ankle sprain for 4 participants, and personal reasons leading 3 participants to discontinue their involvement. A cohort of 40 participants who successfully completed the study in its entirety were randomly divided into two groups (Table 1): the CG (*n* = 20; age = 76.6 ± 5.6 years; BMI = 35.8 ± 2.7 kg/m^2^) and the TG (*n* = 20; age = 74.1 ± 3.7 years; BMI = 35.8 ± 2.7 kg/m^2^).

**Figure 4:**
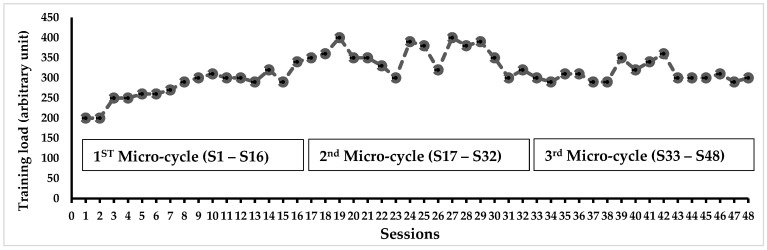
Progression of training load over six months in the trained group. Note: In each session, the training load was calculated using the participant’s RPE after each 60-min session. The individual training load was determined by multiplying the duration of the session by the participant’s RPE score. The overall training load presented in this figure was obtained by summing the individual loads and dividing it by the number of participants.

**Figure 5:**
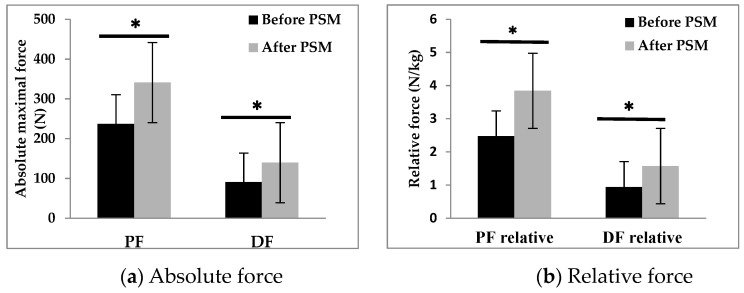
Absolute (**a**) and relative (**b**) forces of plantar and dorsal flexors before and after the PSM program. PF: plantar flexors; DF: dorsal flexors. *: *p* < 0.05, significant difference.

The training load evolution during the PSM program was measured in all participants (Figure 4). The analysis of the Ricci and Gagnon questionnaire revealed that CG (10.9 ± 2.5) and TG (9.7 ± 3.4) were inactive before the PSM program. At the baseline assessment, anthropometric and physical characteristics, walking, and neuromuscular parameters exhibited no significant disparities between the CG and TG. After the PSM program, TG showed a significant increase in LBM (+10%, *p* < 0.05) and a decrease in FBM (−12.4%, *p* < 0.05) (Table 1). GT demonstrated improved physical performance in various exercises including arm flexion (+29.1%, *p* < 0.01), sit-to-stand test (+45.5%, *p* < 0.001), walking back and forth (−32.7%, *p* < 0.01), 2-min walk (+81%, *p* < 0.001), and 2-min knee lifts (+47.3%, *p* < 0.001) (Table 2). The PSM program also resulted in an increase in absolute and relative maximal strength of plantar (+43%, +26%, *p* < 0.05, respectively) and dorsiflexor (+54%, +33%, *p* < 0.05, respectively) muscles (Figure 5).

### 3.2. Gait Parameters

Spatiotemporal parameters, gait cycle parameters, center of pressure displacement parameters, and vertical ground reaction forces are presented in Table 3. Following the PSM program, TG exhibited significant improvements, including a remarkable increase in comfortable walking speed (+80%, *p* < 0.001) and step length (+38%, *p* < 0.05). The duration of the left and right stance phases decreased (−5%, −5%, respectively; *p* < 0.05), while the durations of the left and right swing phases increased (+4.9%, +5%, respectively; *p* < 0.05). Additionally, the durations of the initial left and right double stance phases (−4.1%, −4%, respectively) and the left and right pre-swing phases (−3%, −3%, respectively; *p* < 0.05) decreased. Moreover, TG demonstrated a reduction in CoP velocity (−26%, *p* < 0.01). Notably, TG also displayed increased GRFv in the left (+16%, *p* < 0.05) and right (+14%, *p* < 0.05) P1 forces, as well as the left (+24%, *p* < 0.05) and right (+31%, *p* < 0.05) P2 forces.

### 3.3. EMG Muscle Activity

**Figure 6:**
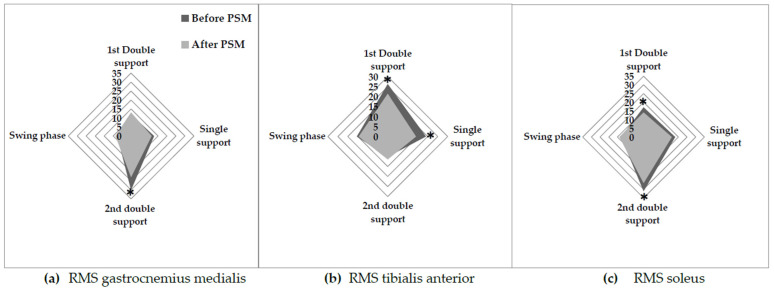
Ankle muscle activity during the phases of the gait cycle before and after PSM program. RMS: root mean square; GM: gastrocnemius medialis; SOL: soleus, TA: tibialis anterior; *: *p* < 0.05 between before and after PSM program.

After the PSM program, GT exhibited decreased GM activity during the second double support phase (−23.9%; *p* < 0.01). Additionally, TA activity decreased during the first double support phase (−18.6%; *p* < 0.05) and single support phase (−26.6%; *p* < 0.01), while muscle activity in SOL decreased during the first stance phase (−17.5%; *p* < 0.05) and second double support phase (−15.1%; *p* < 0.05) (Figure 6).

### 3.4. Pearson’s Correlation Analysis

The person correlation analysis revealed significant correlations between the changes in gait parameters and the evolution of neuromuscular parameters following the PSM program (Table 4). Notably, positive correlations were found between the decrease in CoP velocity and the increase in relative force of PF (r = 0.61, *p* < 0.05) as well as DF (r = 0.63, *p* < 0.05). Furthermore, a positive correlation was observed between the decrease in CoP velocity and the reduction in RMS SOL (r = 0.57, *p* < 0.05) and RMS TA (r = 0.54, *p* < 0.05). Additionally, a positive correlation was identified between the increase in relative P1 and the absolute DF force (r = 0.74, *p* < 0.05). A positive correlation was also found between relative P2 and the increase in the absolute DF force (r = 0.76, *p* < 0.05).

## 4. Discussion

The objective of this study was to evaluate the impact of a physical activity program based on muscle strengthening, balance, and motor exercises on body composition, physical performance, neuromuscular capacities, and biomechanical gait parameters in older adults with SO. The results indicate that the PSM program improved walking ability and functional capacities in this population. These improvements were associated with an improvement in neuromuscular capacities. 

### 4.1. Effect of the PSM Program on Body Composition and Physical Performance

Our study contributes to the existing body of literature [42,46,47] by demonstrating significant improvements in lean body mass (LBM) (+10%) and a decrease in fat body mass (FBM) (−12.4%) among participants in the intervention group after completing the PSM program. However, we did not observe significant impacts on BMI or body weight, which is consistent with previous studies that have explored the effects of different exercise modalities on body composition [42,46,47,60]. The lack of significant changes in BMI and BW can be attributed to the substantial increase in LBM, which replaced FBM. This underscores the importance of considering body composition as a crucial indicator of the effectiveness of exercise programs, particularly in individuals with SO. Focusing solely on changes in BW or BMI may overlook the positive transformations occurring within the body, such as the development of LBM and the reduction of FBM. While some previous studies have also reported similar findings of non-significant changes in BMI and body weight [42,61] it is essential to recognize that the effects of exercise interventions can vary based on factors such as duration, intensity, and individual characteristics. Therefore, the absence of significant changes in these measures should not be interpreted as an indication of ineffectiveness but rather as a reflection of the specific adaptations occurring within the body, primarily the beneficial increase in lean muscle mass. Changing the body composition of individuals with SO presents significant challenges and complexities. It has been observed that individuals with SO may exhibit a blunted response to exercise, which can be attributed to poor insulin sensitivity [62,63]. Poor insulin sensitivity is known to impair muscle protein breakdown and hinder muscle protein synthesis [64]. The presence of obesity often accompanies a chronic inflammatory state and negatively influences the progression [65]. Considering our findings, the effectiveness of our proposed program, including the specific modalities of intensity and frequency, can be considered as a promising approach in addressing these challenges.

Our study demonstrated significant improvements in various measures of physical performance following the implementation of the PSM program (Table 2). Notably, the program led to a substantial increase in the relative maximal force of the plantar (+26%) and dorsal (+33%) flexor muscles. These findings are consistent with a previous study that reported enhanced mobility and functional performance in older adults with SO. Overall, our results suggest that the PSM program has the potential to enhance physical performance in individuals with SO through multiple mechanisms. Firstly, exercise has been shown to stimulate the secretion of insulin-like growth factor 1 (IGF-1), a potent anabolic hormone that promotes muscle maintenance and growth [42,47]. It is plausible that the proposed exercise interventions in our program significantly improve IGF-1 levels, thereby contributing to the observed improvements in muscle mass and function. Secondly, the improvements in physical performance observed in our study are likely associated with the increase in strength and lean body mass [44,66,67].

### 4.2. Effect of the PSM Program on Ankle Muscle Strength and Neuromuscular Strategies during Walking

To our knowledge, this study is the first to evaluate the effects of a physical activity program on neuromuscular capacities and their contribution to the improvement of walking abilities in older adults with SO. The results of this study showed that walking parameters, including preferred gait speed (+66%), maximal gait speed (+71%), and step length (+38%) in SO older adults were significantly improved after the PSM program and were associated with a reduction in the duration of the stance phase (−5%) and an increase in the duration of the swing phase (+4.9%). These improvements can be attributed to two possible mechanisms. First, the improvements in gait parameters could be attributed to a reduction in postural instability during walking. Maktouf et al. [34] demonstrated that obese older adults exhibited higher CoP velocity and increased activity of TA during walking. The TA muscle plays a significant role in amortizing body mass during the first double support phase of walking. Interestingly, age appears to be a stronger predictor of increased TA activity in obese older adults [34]. This adaptive neuromuscular response, which may serve as a compensatory mechanism, can be explained by age-related muscle postural control and somatosensory system alterations [68]. The inclusion of specific balance exercises in our PES program likely played a crucial role in enhancing the participants’ balance capacities and stability during walking. This can be supported by the positive correlation observed between the decrease of CoP velocity and the SOL (r = 0.57) and TA (r = 0.54) activities among individuals with SO following the program. The decrease in CoP velocity indicates improved postural control, while the reduction in TA activity suggests a more efficient utilization of muscle during walking. These outcomes further underscore the positive effects of the PES program on enhancing balance and stability, ultimately contributing to the improvements in gait among individuals with SO.

Secondly, the improvements in gait parameters observed in our study may be attributed to the enhanced neuromuscular capacities of the ankle muscles. Results showed a positive correlation between the increase of the absolute PF and DF forces and the relative P2 (r = 0.76) and P1 (r = 0.74) in the TG. The increase in GRFv during walking indicates improved force generation during the amortization (P1) and propulsion (P2) phases of walking. Notably, this increase in force generation was accompanied by a decrease in muscle activity of the GM (−23.9%) and SOL (−17.5%) during the first stance phase and propulsive phase, respectively. These findings suggest that the augmented maximal force production capacity of the plantar flexor muscles, achieved through muscle strengthening exercises, partially contributed to the enhancement of gait quality in older adults with SO. Specifically, obese older adults face difficulties in generating adequate force relative to their body mass during forward propulsion [34]. Consequently, they tend to increase GM and SOL muscle activity during the propulsive phase of gait to manage the propulsive movement of their greater body mass and stabilize the excessive body mass during forward propulsion [34]. The decrease in muscle activity was also observed in SOL during the first stance phase, where it acts as an antagonist muscle. This finding suggests a reduction in muscle coactivation among obese elderly individuals, which may contribute to a decrease in energy expenditure during walking [69,70]. By minimizing the coactivation of muscles during the first stance phase, individuals can optimize their movement efficiency and potentially reduce the metabolic demands associated with walking [71]. Considering these observations, it can be inferred that the PSM program improved force production capacities through targeted strengthening exercises designed to counter the mechanical constraints imposed by obesity, such as mobilizing a significant body mass and addressing the sarcopenia-associated decline in force production.

Furthermore, it is crucial to recognize that the observed improvement in force production capacity, specifically the increased muscle strength of the ankle muscles, did not demonstrate a direct correlation with the enhancement of gait parameters, such as maximal speed and step length. This finding highlights the multifaceted nature of walking and suggests that solely focusing on developing greater muscle strength or improving postural control stability may not be sufficient to enhance the overall quality of walking [72]. Walking involves a coordinated integration of various factors, including muscle strength, joint mobility, and postural control [73]. While muscle strength is undoubtedly important for generating force during walking, other factors, such as joint range of motion, also play a critical role in determining walking capacity [73,74]. Joint mobility, particularly at the ankle joint, allows for a fluid and efficient stride, contributing to stride length and overall walking mechanics [72,75]. Therefore, it is plausible to consider that the motor exercises incorporated into the PES program, which specifically targeted joint range of motion and lower limb mobility, made significant contributions to the observed improvements in walking parameters. By addressing joint mobility and lower limb flexibility, these exercises likely facilitated a more optimal range of motion at the ankle joint, enabling smoother and more efficient gait patterns. This, in turn, could have positively influenced walking parameters such as stride length and step cadence.

### 4.3. Practical Recommendations

Based on the findings of our study, the implementation of a physical activity program for frail populations, such as older adults with SO, should prioritize two key aspects. Firstly, it is crucial to tailor the quality of exercise to address the specific characteristics and impairments of each individual. This personalized approach is essential to directly target the identified alterations and promote effective interventions. Secondly, the quantitative aspect of the program should be based on patients’ perceived exertion, allowing for the prescription of an optimal exercise intensity for each individual. This approach ensures that patients do not work under fatigued conditions, which may yield negative outcomes. Moreover, it enables the provision of an appropriate intensity that adequately challenges the targeted systems, preventing the use of inefficient dosages that fail to elicit the necessary stimulation for adaptation. Furthermore, regular assessments throughout the program are essential to monitor progress and make necessary adjustments to the training load or exercise type, facilitating ongoing adaptation and optimal outcomes. Finally, it is vital to consider psychological and social factors, such as motivation, to ensure the continuity of physical activity practice among older adults. By creating a supportive and engaging environment, the program can foster sustained motivation, thereby promoting adherence and the continued benefits of physical activity for older adults with SO.

### 4.4. Limitations and Perspectives

This study presents a pioneering investigation into the effects of a physical activity program focused on muscle strengthening, posture, and motor exercises on gait parameters and neuromuscular strategies during walking in older adults with SO. This innovative approach provides valuable insights into the underlying mechanisms that contribute to the observed improvements in this population. However, there are several limitations to consider. Firstly, the small sample size may limit the generalizability of our findings. Future studies with larger sample sizes are needed for validation and increased statistical power. Furthermore, our study concentrated mainly on the muscles surrounding the ankle joint. Given the potential for excess adipose tissue in the areas of the knee and hip joints to influence EMG signals, it would be advantageous to incorporate a 3D gait analysis. This would involve assessing joint moments and ranges of motion across all three joints—hip, knee, and ankle. Moreover, dietary intake was not monitored, but future studies could explore the synergistic effects of physical activity and protein intake. Finally, comorbidities and specific medical conditions may have influenced outcomes, suggesting the need for adjustment in future studies.

## 5. Conclusions

This study demonstrated that a physical activity program incorporating muscle strengthening, balance, and motor exercises had a positive impact on older adults with SO. The program effectively improved body composition, physical performance, gait, and neuromuscular capacities in this population. The improvement in walking ability can be attributed to the reduction of age- and obesity-related impairments. Despite the acute effects of aging and obesity, this study confirmed the trainability of neuromuscular capacities in older adults with SO, highlighting the potential for reversibility and improvement. Considering the reversibility of neuromuscular impairments, it is crucial to promote early intervention and sustained engagement in physical activity to maximize the potential for long-term improvements in mobility and independence. By integrating these findings into clinical practice and public health strategies, we can work towards optimizing the health and well-being of older adults with SO.

## Figures and Tables

**Table 1:** Comparison of physical and anthropometric characteristics between the control and trained groups at the baseline and after the intervention.

	CG	TG	*p*	CG	TG	*p*
	At the Baseline			After the Intervention		
**Characteristics**	Anthropometric parameters					
Age (years)	76.6 ± 5.6	74.1 ± 3.7	NS	77.1 ± 5.6	74.6 ± 3.7	NS
Body height (cm)	160.3 ± 4.5	164.5 ± 5.5	NS	160.3 ± 4.5	164.5 ± 5.5	NS
Body mass (kg)	91.7 ± 6.7	95.9 ± 4.3	NS	93.7 ± 5.7	88.7 ± 5.7	NS
BMI (kg/m^2^)	35.8 ± 2.7	35.8 ± 2.1	NS	36.6 ± 2.7	34.6 ± 2.7	NS
Body fat (%)	42.1 ± 4.5	43.0 ± 3.5	NS	44.3 ± 7.5	34.2 ± 2.5 *	*p* < 0.05
FBM (kg)	38.6 ± 3.8	41.2 ± 3.3	NS	41.6 ± 3.8	30.3 ± 4.1 *	*p* < 0.05
LBM (kg)	53.1 ± 4.4	54.7 ± 5.4	NS	52.1 ± 4.4	58.4 ± 4.4 *	*p* < 0.05
Hip circumference (cm)	96.8 ± 6.8	96.8 ± 6.8	NS	96.8 ± 6.4	94.8 ± 2.8	NS
Waist circumference (cm)	99.8 ± 5.1	99.8 ± 6.1	NS	99.8 ± 5.1	97.8 ± 4.1	NS
Handgrip force (N)	13.5 ± 3.1	12.9 ± 2.4	NS	13.1 ± 2.9	14.1 ± 3.2 *	*p* < 0.05
Maximal gait speed (m/s)	0.7 ± 0.2	0.7 ± 0.2	NS	0.8 ± 0.2	1.2 ± 0.3 *	*p* < 0.05
**Tests**	Senior fitness test					
30 s—Arm flexion (n)	18.2 ± 3.9	17.3 ± 3.9	NS	19.2 ± 4.9	23.5 ±5.4 *	*p* < 0.05
30 s—Sit to stand (n)	10.1 ± 3.3	11.1 ± 3.3	NS	9.4 ± 2.3	14.7 ± 3.5 *	*p* < 0.05
Trunk flexibility (cm)	12.4 ± 9.9	11.4 ± 7.8	NS	11.4 ± 7.9	7.0 ± 5.4	NS
Walking back and forth (s)	21.1 ± 3.9	20.5 ± 3.9	NS	20.1 ± 4.9	14.2 ± 4.5 *	*p* < 0.05
Upper body flexibility (cm)	26.4 ± 8.1	27.4 ± 7.9	NS	26.1 ± 8.7	22.0 ± 9.8 *	*p* < 0.05
2 min—Walk (m)	72.3 ± 20.1	75.5 ± 21.4	NS	72.3 ± 19.5	131.0 ± 45.2 *	*p* < 0.05
2 min—Knee lifts (n)	82.4 ± 55.5	86.5 ± 56.3	NS	77.4 ± 47.5	121.4 ± 62.1 *	*p* < 0.05

CG: control group; TG: trained group; BMI: body mass index; FBM: fat body mass; LBM: lean body mass; *: *p* < 0.05, difference between groups; NS: no significant difference.

**Table 2:** Senior fitness test results of the trained group before and after PSM program.

Tests	Before PSM	After PSM	Ϫ (%)	*p*
30 s—Arm flexion (n)	17.3 ± 3.9	23.5 ± 5.4 *	+29.1	*p* < 0.01
30 s—Sit to stand (n)	11.1 ± 3.3	14.7 ± 3.5 *	+45.5	*p* < 0.001
Trunk flexibility (cm)	11.4 ± 7.8	7.0 ± 5.4	−32.7	NS
Walking back and forth (s)	20.5 ± 3.9	14.2 ± 4.5 *	−16.0	*p* < 0.001
Upper body flexibility (cm)	27.4 ± 7.9	22.0 ± 9.8 *	−12.4	*p* < 0.05
2 min—Walk (m)	75.5 ± 21.4	131.0 ± 45.2 *	+81.2	*p* < 0.001
2 min—Knee lifts (n)	86.5 ± 56.3	121.4 ± 62.1 *	+47.3	*p* < 0.001

TG: trained group; n: number of repetitions; Ϫ: difference between before and after PSM program; *: significant difference between before and after PSM program; NS: no significant difference.

**Table 3:** Gait parameters of the trained group before and after the PSM program.

	TG			
Gait Parameters	Before PSM	After PSM	Ϫ (%)	*p*
Preferred gait speed (m/s)	14.2 ± 4.5 *	1.0 ± 0.2 *	+66	*p* < 0.001
Step length (cm)	14.2 ± 4.5 *	33.1 ± 4.2 *	+38	*p* < 0.01
Walking cycle				
Support phase (%) Left	78.3 ± 2.8	73.3 ± 1.8 *	−5.0	*p* < 0.05
Support phase (%) Right	77.5 ± 3.8	72.5 ± 2.8 *	−5.0	*p* < 0.05
1st double support (%) Left	26.5 ± 5.2	26.8 ± 2.2 *	−4.1	*p* < 0.05
1st double support (%) Right	25.3 ± 4.0	21.3 ± 2.0 *	−4.0	*p* < 0.05
Single support (%) Left	24.8 ± 5.9	26.8 ± 2.9	-	-
Single support (%) Right	25.4 ± 6.1	27.3 ± 3.1	-	-
2nd double support (%) Left	27.0 ± 3.1	24.0 ± 2.1 *	−3.0	*p* < 0.05
2nd double support (%) Right	26.9 ± 3.7	23.9 ± 2.7 *	−3.0	*p* < 0.05
Swing phase (%) Left	21.8 ± 2.8	26.7 ± 1.9 *	+4.9	*p* < 0.05
Swing phase (%) Right	22.5 ± 3.8	27.5 ± 2.8 *	+5.0	*p* < 0.05
CoP parameters				
Maximal speed (cm/s)	158.5 ± 26.5	117.7 ± 21.4 *	−26.0	*p* < 0.01
GRFv parameters				
Relative P1 (N/kg) Left	9.4 ± 1.5	10.9 ± 1.6 *	+16.0	*p* < 0.05
Relative P1 (N/kg) Right	9.5 ± 1.4	10.8 ± 1.2 *	+14.0	*p* < 0.05
Relative P2 (N/kg) Left	9.3 ± 1.2	11.5 ± 1.8 *	+24.0	*p* < 0.05
Relative P2 (N/kg) Right	9.0 ± 1.5	11.8 ± 1.9 *	+31.0	*p* < 0.05

CG: control group; TG: trained group; P1: the 1st peak force; P2: the second peak force; *: difference between before and after PSM program.

**Table 4:** Pearson’s correlation analysis.

	Maximal Voluntary Contraction		Muscle Activity			Body Composition	
Gait Parameters	Ϫ Absolute PF Force	Ϫ Absolute DF Force	Ϫ RMS GM	Ϫ RMS SOL	Ϫ RMS TA	Ϫ LBM	Ϫ FM
Ϫ Maximal gait speed	0.23	0.25	0.21	0.24	0.24	0.11	0.25
Ϫ Step length	0.36	0.29	0.13	0.27	0.28	0.15	0.19
Ϫ CoP velocity	0.61 *	0.63 *	0.17	0.57 *	0.54 *	0.11	0.09
Ϫ Relative P1	0.11	0.74 *	0.15	0.14	0.19	0.25	0.33
Ϫ Relative P2	0.76 *	0.13	0.21	0.27	0.12	0.17	0.21

P1: the first peak force; P2: the second peak force; CoP: center of pressure; LBM: lean body mass; FBM: fat body mass; GM: gastrocnemius medialis; SOL: soleus; TA: tibialis anterior; Ϫ: difference between before and after PSM program; *: *p* < 0.05.

## Data Availability

The data supporting the reported results of this study will be published on the Pan African Clinical Trials Registry after the publication of the article. Interested parties can access the data by referring to the registry once it becomes available.
